# Optimal method for assessment of respiratory muscle strength in neuromuscular disorders using sniff nasal inspiratory pressure (SNIP)

**DOI:** 10.1371/journal.pone.0177723

**Published:** 2017-05-16

**Authors:** Marta Kaminska, Francine Noel, Basil J. Petrof

**Affiliations:** 1 Respiratory Division & Sleep Laboratory, McGill University Health Centre, Montreal, Quebec, Canada; 2 Respiratory Epidemiology and Clinical Research Unit, McGill University Health Centre, Montreal, Quebec, Canada; 3 Translational Research in Respiratory Diseases Program, Research Institute of the McGill University Health Centre, Montreal, Quebec, Canada; 4 Meakins Christie Laboratories, McGill University, Montreal, Quebec, Canada; Universitatsklinikum Freiburg, GERMANY

## Abstract

**Background:**

The ability to accurately determine respiratory muscle strength is vitally important in patients with neuromuscular disorders (NMD). Sniff nasal inspiratory pressure (SNIP), a test of inspiratory muscle strength, is easier to perform for many NMD patients than the more commonly used determination of maximum inspiratory pressure measured at the mouth (MIP). However, due to an inconsistent approach in the literature, the optimal technique to perform the SNIP maneuver is unclear. Therefore, we systematically evaluated the impact of performing the maneuver with nostril contralateral to the pressure-sensing probe open (SNIP_OP_) versus closed (SNIP_CL_), on determination of inspiratory muscle strength in NMD patients as well as control subjects with normal respiratory muscle function.

**Methods:**

NMD patients (n = 52) and control subjects without respiratory dysfunction (n = 52) were studied. SNIP_OP,_ SNIP_CL_, and MIP were measured during the same session and compared using ANOVA. Agreement and bias were assessed with intraclass correlation coefficients (ICC) and Bland-Altman plots.

**Results:**

Mean MIP values were 58.2 and 94.0 cmH2O in NMD and control subjects, respectively (p<0.001). SNIP_CL_ was greater than SNIP_OP_ in NMD (51.9 ±31.0 vs. 36.9 ±25.4 cmH_2_O; p<0.001) as well as in controls (89.2 ±28.1 vs. 69.2 ±29.2 cmH_2_O; p<0.001). In both populations, the ICC between MIP and SNIP_CL_ (NMD = 0.78, controls = 0.35) was higher than for MIP and SNIP_OP_ (NMD = 0.53, controls = 0.06). In addition, SNIP_CL_ was more often able to exclude inspiratory muscle weakness than SNIP_OP_.

**Conclusions:**

SNIP_CL_ values are systematically higher than SNIP_OP_ in both normal subjects and NMD patients. Therefore, SNIP_CL_ is a useful complementary test for ruling out inspiratory muscle weakness in individuals with low MIP values.

## Introduction

Accurate assessment of respiratory muscle strength is clinically important in patients with neuromuscular disorders (NMD) or unexplained dyspnea. Measurement of maximum inspiratory pressure (MIP) at the mouth is the most commonly employed test to evaluate inspiratory muscle function, as it is non-invasive and relatively convenient. However, particularly in patients with NMD, MIP suffers from the possible occurrence of falsely low values [1, 2] due to difficulties in maintaining an effective mouth seal or sustaining a maximal inspiratory effort [3]. For these reasons, sniff nasal inspiratory pressure (SNIP) has been used as an alternative non-invasive test of inspiratory muscle function which is easier to perform for many NMD patients [4, 5]. It can be employed to monitor inspiratory muscle strength over time in NMD, and a normal SNIP can also effectively rule out inspiratory weakness in individuals with spuriously low MIP values [4, 6–8]. In amyotrophic lateral sclerosis (ALS), the SNIP has been reported as the best prognostic indicator [9].

The SNIP measurement entails brief maximal sniff efforts by the patient during simultaneous intranasal pressure recordings within a nostril that is sealed by a snugly fitting plug containing the pressure-sensing probe. The sniff maneuver has long been used in the assessment of diaphragm function. It was initially described for radiological assessment of unilateral diaphragm weakness [10]. Subsequently, sniffs were found to be a representative approximation of phrenic stimulation in studies of diaphragm contraction [11]. The sniff maneuver is now commonly used when diaphragm strength is being assessed by measuring transdiaphragmatic pressure (Pdi) [12]. The SNIP, in turn, has been devised as a less invasive alternative.

In the original description of SNIP measurements, the contralateral nostril remained unobstructed or open (henceforth referred to as SNIP_OP_) [4]. Under these conditions, a reliable SNIP_OP_ value presumably requires inspiratory collapse of the contralateral nasal valve [13] in order to allow for quasi-equilibration of intrathoracic and nasal cavity pressures. The SNIP can also be measured as a static maneuver with the contralateral nostril closed (SNIP_CL_), as reported in a much smaller number of studies [14, 15]. Although they appear to be used and reported interchangeably in the literature, it is unclear if SNIP_OP_ and SNIP_CL_ in fact produce the same results. Theoretically, they could be very similar in healthy subjects, but individuals with inspiratory muscle weakness may be unable to generate sufficient negative inspiratory pressure to collapse the nasal valve when performing SNIP_OP_ [13]. Therefore, SNIP_OP_ might poorly reflect the actual negative intrathoracic pressure values and thus provide inaccurate information about the true level of inspiratory muscle strength in some NMD patients.

In the present study, we sought to determine whether there are any systematic or clinically significant differences between values of SNIP_OP_ and SNIP_CL_ in patients with known NMD as well as in patients without clinical evidence of respiratory dysfunction. Our primary hypothesis was that values of SNIP_CL_ would be significantly higher than SNIP_OP_ in NMD patients. The secondary hypothesis was that in patients with a reduced MIP value, SNIP_CL_ would give results within a normal range (suggesting an absence of respiratory muscle weakness) more often than SNIP_OP_.

## Materials & methods

### Study subjects

The two groups of study subjects consisted of: 1) NMD patients recruited from a home non-invasive ventilation program, and 2) a control group comprised of individuals with obstructive sleep apnea (OSA) and no known NMD or significant lung disease who were also participants in a Pompe disease screening study. The NMD diagnosis was obtained from the medical record, as made by a clinical neurologist. Two patients in the initial control group were identified as having a NMD and thus transferred into the NMD group. In addition, several control group patients (n = 8 with asthma or chronic obstructive pulmonary disease (COPD), n = 5 with morbid obesity, n = 7 with other) had abnormal spirometry (FEV1 or FVC < 80% of predicted, or FEV1/FVC ratio <70%) and were thus excluded.

Subjects were recruited between September 2013 and November 2014, and provided their written informed consent. Participants, none of whom were hospitalized at the time of testing, took their usual medications without modification. All study subjects underwent spirometry and respiratory muscle strength measurements as outlined below, all in the sitting position, and all within a single testing session between 10 am and 2 pm. The study was approved by the institutional Research Ethics Board of the McGill University Health Center (13-379-BMB).

### SNIP measurements

Both SNIP_OP_ and SNIP_CL_ were performed using a commercially available device (MicroRPM, VIASYS Healthcare, Hochberg, Germany) with disposable nasal probes. Factory-set calibration of the device was verified using a manometer. The nostril that appeared most patent clinically was chosen for insertion of the nasal probe and the appropriate nasal probe size was verified by ensuring the absence of air leak during sniffs. Without a prior training period, the patient was asked to perform short, sharp sniffs of maximal intensity from functional residual capacity (FRC) in the sitting position with the mouth closed. Normal breathing was allowed between trials. At least 10 trials were done in total: five sniffs with contralateral nostril open (SNIP_OP_), and five with the contralateral nostril closed (SNIP_CL_). Half of the participants performed SNIP_OP_ first, whereas the reverse order was used in the other half, to account for any potential learning or order effect. The highest value for each SNIP method is reported for each individual. A single research assistant performed all testing.

### Standard PFT measurements

Spirometry was performed (Jaeger FlowScreen V2.6.0, Carefusion Corp, San Diego, CA) to determine forced expiratory volume in one second (FEV1), forced vital capacity (FVC), and peak expiratory flow (PEF) according to ATS guidelines [16] and established reference values [17]. Supine spirometry was subsequently similarly performed. Wheelchair-bound participants who could not easily transfer did not have supine measurements, unless their wheelchair tilted to at least a 30 degree recline. MIP was measured through a flanged mouthpiece from residual volume (RV) [12]. The highest of at least three consistent values was recorded as recommended. Reference values were taken from Vincken et al. [18]. Individuals with values reaching the upper saturation limit of the MIP manometer (≥150 cmH_2_O) were excluded from the analysis (n = 5 from the control group) to avoid a ceiling effect which could introduce error into the analyses.

### Statistical analysis

Unpaired t-tests were used for comparisons of baseline characteristics between NMD and control groups. Descriptive statistics are presented as mean and standard deviation (SD), unless specified otherwise. Normality of outcomes data was tested using the Shapiro-Wilk test. Control data were normally distributed but not NMD data. Therefore, we used ANOVA to compare the SNIP_OP_, SNIP_CL_ and MIP measurements in controls, and (nonparametric) Friedman ANOVA for the NMD group. Intraclass correlation coefficients (ICC) were performed for combinations of SNIP_OP_, SNIP_CL_ and MIP within groups. Scatter plots and Bland-Altman plots were generated, and bias was defined as the mean of the differences between two measurement values. Limits of agreement were calculated as bias +/- (1.96 x SD for the difference). The Fisher exact test was used to compare counts. Statistical significance is defined as p<0.05. Analyses were done using SAS software, version 9.3.

#### Power calculation

Our sample size was based on a detectable difference between SNIP_CL_ and SNIP_OP_ of 10 cmH_2_O, which we considered the minimum that would be relevant, and assumed a normal distribution. For a sample size of 50 patients (in each group separately), using a paired t-test and conservative estimate for the standard deviation of the difference of 20 cmH_2_O, we would have a power of 93% to detect a difference of 10 cmH_2_O with type I error of 0.05.

## Results

Table 1 shows demographic and PFT data for the 52 NMD patients and 52 control subjects included in the study. The two groups did not differ with respect to age, although the control group tended to include more females and had a higher average body mass index. NMD patients demonstrated mild to moderate reductions in spirometric values, which were significantly lower than the control group (73.1% vs. 98.6% of predicted for FVC, p<0.001).

**Table 1:** Demographic and pulmonary function characteristics of study patients.

Mean (SD)	Neuromuscular patients (n = 52)	Control subjects (n = 52)	p
Age (yrs)	51.0 (16.9)	51.7 (12.3)	0.55
Sex (% males)	50.0%	26.9%	0.03
BMI (kg/m^2^)	26.8 (6.42)	31.0 (5.7)	< 0.001
Neuromuscular disorder diagnoses (n)			
Post-polio syndrome	9		
Myotonic muscular dystrophy	5		
Fascio-scapulo-humeral dystrophy	4		
Duchenne muscular dystrophy	2		
Other muscular dystrophies (Becker, Occulopharyngeal, Emery Dreyfuss, Limb girdle)	4		
Phrenic palsy	5		
ALS	4		
Charcot Marie Tooth disease	2		
Pompe disease	2		
Other	15		
FEV_1_ (L)	1.94 (0.99) (n = 49)	2.63 (0.80)	< 0.001
FEV_1_ (% of pred)	69.0 (28.5) (n = 49)	93.1 (12.3)	< 0.001
FVC (L)	2.46 (1.24) (n = 49)	3.32 (1.04)	0.002
FVC (% of pred)	73.1 (30.1) (n = 49)	99.0 (12.5)	< 0.001
FEV_1_/FVC (%)	76.1 (17.3) (n = 49)	84.7 (5.7)	<0.001
PEF (L/min)	5.00 (2.17) (n = 49)	5.65 (1.77) (n = 47)	0.006
PEF (% pred)	69.5 (27.0) (n = 49)	82.4 (17.9) (n = 47)	< 0.001
Change in FEV_1_ in supine position (%)	-14.6 (14.8) (n = 35)	-6.3 (8.7) (n = 51)	< 0.001
Change in FVC in supine position (%)	-11.5 (14.8) (n = 35)	-0.2 (6.0) (n = 51)	< 0.001
SaO_2_ (%)	96.8 (1.67) (n = 51)	97.4 (1.45)	0.047

BMI: body mass index; ALS: amyotrophic lateral sclerosis; FEV_1_: forced expiratory volume in 1 second; FVC: Forced vital capacity; PEF: Peak expiratory flow; SaO_2_: hemoglobin oxygen saturation (measured by pulse oximetry with a finger probe).

**Fig 1:**
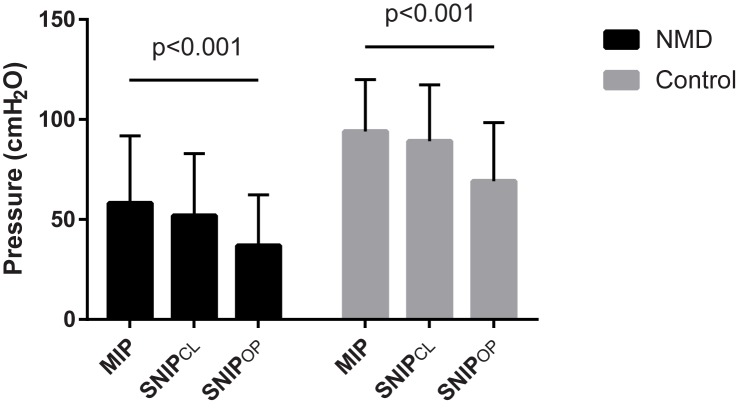
Group mean values for MIP and the two SNIP methods in NMD patients and control subjects. Mean SNIP_OP_ was significantly lower than SNIP_CL_ and MIP in both groups (ANOVA, see methods). The error bar represents the standard deviation.

**Fig 2:**
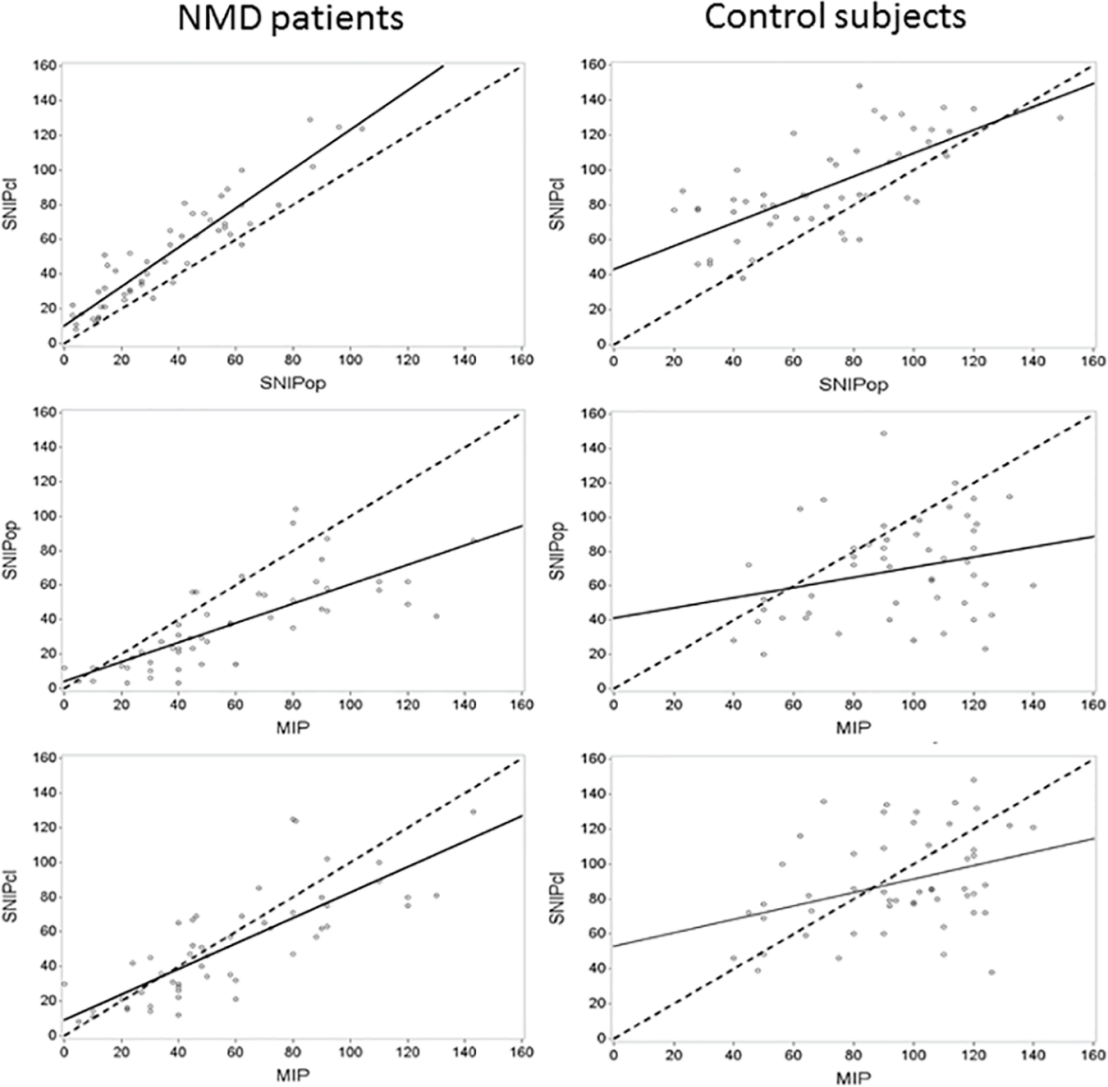
Scatter plots of relationships between the two SNIP methods and MIP in individual NMD patients and control subjects. The dashed line represents the identity line; the solid line is the correlation line. There are generally good correlations between pairs of values in NMD patients, whereas more scatter is seen in the control group and at higher values in the NMD group. The SNIP_CL_ values are systematically higher than SNIP_OP_, especially in the NMD group.

Fig 1 shows MIP, SNIP_OP_ and SNIP_CL_ values in the control and NMD groups. As expected, all values were significantly lower in NMD compared with control subjects (p<0.001). Neither age nor sex correlated with SNIP_CL_ values in the control and NMD groups, whereas age was weakly correlated with SNIP_OP_ (r = 0.295, p = 0.03) in the NMD group only. The mean SNIP_OP_ value was significantly lower than SNIP_CL_ and MIP in both groups. Results were identical irrespective of the order in which SNIP_OP_ and SNIP_CL_ were performed (results not shown). Scatter plots demonstrating the relationships between these parameters in individual patients are shown in Fig 2.

**Fig 3:**
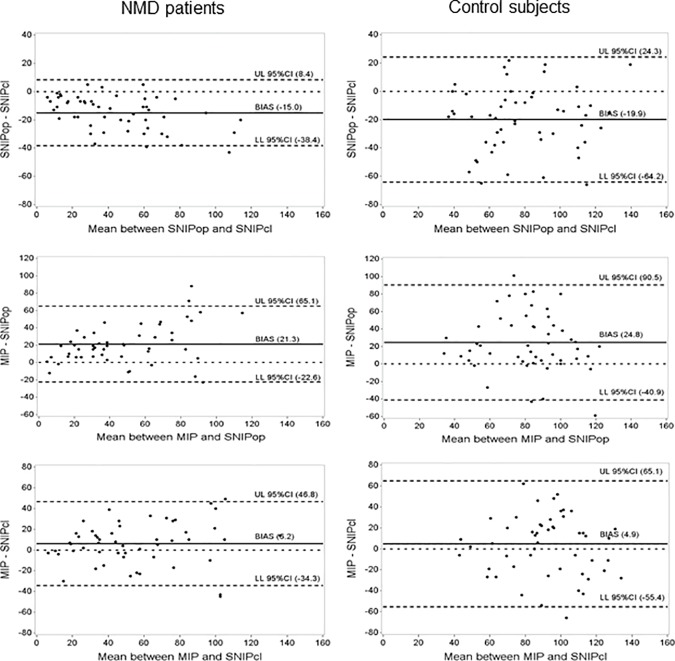
Bland-Altman plots indicating agreement and bias between the two SNIP methods and MIP in NMD patients and control subjects. UL 95% CI: upper limit of 95% confidence interval; LL 95% CI: lower limit of 95% confidence interval. Visual inspection reveals that SNIP_CL_ is greater than SNIP_OP_ on average, and both are lower than the MIP. The biases are similar between groups for pairs of measurements, but limits of agreement are wider in the control group. Agreement is generally better at lower values in both groups.

To assess agreement between measurements, ICC was calculated between SNIP_OP,_ SNIP_CL_ and MIP (Table 2). Agreement was poorer in the control subjects than in the NMD group for all combinations of measures. In both groups the highest agreement was for SNIP_OP_ vs. SNIP_CL_, and SNIP_CL_ was in better agreement with MIP than SNIP_OP_. Agreement and bias were further assessed using Bland-Altman plots (Fig 3). These plots indicate that SNIP_CL_ is greater than SNIP_OP_ for the majority of subjects, with a mean bias of -15.04 in NMD and -19.9 in controls. Moreover, the bias between SNIP_CL_ and MIP was substantially lower (consistent with better agreement) than between SNIP_OP_ and MIP. This was true for both NMD and control groups, although the limits of agreement are narrower (less scatter) for NMD compared with control subjects. Visual inspection of the plots also suggests less scatter at lower values, particularly in NMD patients.

**Table 2:** Intraclass correlation coefficients (ICC) comparing the 3 measurements of inspiratory muscle strength.

	Neuromuscular patients		Control subjects	
	n = 52		n = 52	
	ICC	95% CI	ICC	95% CI
SNIP_OP_ vs. SNIP_CL_	0.79	0.66–0.87	0.51	0.28–0.68
MIP vs. SNIP_OP_	0.53	0.31–0.70	0.06	-0.21–0.32
MIP vs. SNIP_CL_	0.78	0.65–0.87	0.35	0.09–0.56

ICC: Intraclass correlation coefficient. CI: Confidence intervals.

To assess whether SNIP_CL_ might be more useful clinically than SNIP_OP_ to rule out inspiratory muscle weakness in subjects with reduced MIP, we determined how often SNIP_OP_ or SNIP_CL_ were higher than MIP in individuals with a low MIP value, as determined using three different thresholds for MIP (Table 3). For MIP < 80% of predicted, SNIP_CL_ was higher than MIP more frequently than SNIP_OP_ in NMD patients (40% vs. 14%, p = 0.03). For MIP < 60% of predicted, SNIP_CL_ was also more often higher than MIP as compared to SNIP_OP_ in NMD patients (48% vs. 10%, p = 0.02). In control subjects, MIP was < 80% of predicted in 4 subjects and < 60% of predicted in 1 subject. The latter subject had both SNIP_OP_ and SNIP_CL_ higher than MIP, while another control subject had a SNIP_CL_ (but not SNIP_OP_) higher than MIP. Finally, we assessed subjects with MIP <80 cmH_2_O, selected because this represents a threshold value above which clinically significant inspiratory muscle weakness is considered to be highly unlikely [12]. SNIP_CL_ and SNIP_OP_ were higher than MIP in 44% and 14% of 36 NMD patients with MIP falling below this threshold, respectively (p = 0.003). In controls, this occurred in 69% vs. 31% of 13 subjects, respectively (p = 0.12).

**Table 3:** Comparison of the two SNIP methods in patients with reduced MIP.

	MIP < 80% of predicted			MIP < 60% of predicted			MIP < 80 cmH_2_O		
	SNIP_OP_ > MIP	SNIP_CL_ > MIP	p	SNIP_OP_ > MIP	SNIP_CL_ > MIP	p	SNIP_OP_ > MIP	SNIP_CL_ > MIP	p
**NMD**	**(n = 35)**			**(n = 21)**			**(n = 36)**		
n (%)	5 (14%)	14 (40%)	0.03	2 (10%)	10 (48%)	0.02	5 (14%)	16 (44%)	0.003
Mean difference(range, cmH_2_O)	10.2 (2–16)	13.9 (1–45)	0.40	7.0 (2–12)	10.2 (1–30)	0.37	7.6 (2–12)	11.3 (1–30)	0.27
**Control**	**(n = 4)**			**(n = 1)**			**(n = 13)**		
n (%)	1 (25%)	2 (50%)	0.99	1 (100%)	1 (100%)	-	4 (31%)	9 (69%)	0.12
Mean difference(range, cmH_2_O)	43 (-)	30 (6–54)	-	43 (-)	54 (-)	-	28 (2–43)	29.7 (6–66)	0.89

Lastly, we evaluated how often SNIP_OP_ or SNIP_CL_ values fell within the normal range in subjects with reduced MIP. The recommended lower limit of normal (LLN) for SNIP is 70 cmH_2_O for males, and 60 cmH_2_O for females [12]. In NMD patients with MIP< 80 cmH_2_O, 1 subject had a SNIP_CL_ value > LLN, whereas this did not occur for SNIP_OP_ in any NMD patient. In controls with MIP < 80 cmH_2_O, 3 subjects had both SNIP_OP_ and SNIP_CL_ values > LLN, while 4 had only SNIP_CL_ > LLN and none had only SNIP_OP_ > LLN.

## Discussion

Although MIP is the most widely used test of inspiratory muscle strength in standard clinical practice, it is clear from previous work that the use of a single test such as MIP tends to overdiagnose weakness [2]. SNIP has thus been recommended as a complementary test to help address this issue, particularly in NMD patients [1, 6, 7]. However, SNIP_OP_ and SNIP_CL_ have been utilized in a seemingly interchangeable fashion by different investigators to assess inspiratory muscle strength [2, 7, 14, 15]. Moreover, very few studies in the literature have actually reported on the use of SNIP_CL_ to evaluate inspiratory muscle function [14, 15]. To our knowledge, this is the first study to formally compare the two methods of SNIP measurement in NMD patients as well as control subjects with normal inspiratory muscle strength. Our main findings are that SNIP_CL_ values are systematically greater than SNIP_OP_ in both NMD and controls, and that the level of agreement with MIP is also superior for SNIP_CL_ in comparison to SNIP_OP_. Therefore, in patients with a low MIP value, SNIP_CL_ appears to be a more useful test than SNIP_OP_ for excluding inspiratory muscle weakness.

Both MIP and SNIP have a learning effect and are operator-dependent [19, 20], but several aspects of SNIP may be more advantageous in NMD patients [3]. The SNIP requires only a short burst of maximal inspiratory muscle contraction, whereas the MIP involves sustaining a maximal inspiratory effort for at least 1 second. This more prolonged effort required for MIP may be difficult for some patients, resulting in falsely low values. Furthermore, in principle SNIP can be performed in individuals who are unable to maintain a tight lip seal around a mouthpiece, which is frequently the case in NMD. The maneuver required for SNIP is also generally regarded as more natural and easier to explain to patients [21]. In keeping with the above, SNIP was reported to be more predictive of outcomes than MIP in ALS [9] and Guillain-Barré syndrome [22].

SNIP_OP_ has been reported to be higher than MIP in some studies [23, 24], and this appears to be more prevalent in those individuals with the least amount of weakness [7, 8]. Conversely, Hart et al. [25] found, in a group of NMD patients, that MIP was greater than SNIP_OP_ (4.8 cmH2O bias with both tests performed from FRC), and SNIP_OP_ was lower as a proportion of MIP in those patients with the most severe impairment [25]. We speculate that the above findings are at least partly explained by an inability of very weak patients to generate a sufficiently negative inspiratory pressure to collapse the nasal valve within the open nostril during SNIP_OP_ measurements [26]. In contrast, by occluding the nostril during SNIP_CL_, the measurement becomes a static one such that pressures are more readily equilibrated throughout the airways.

It is interesting to note that SNIP_OP_ (but not SNIP_CL_) was also lower than MIP in the control subjects of our study, suggesting that additional factors other than weakness are involved in the better equilibration of pressures achieved with SNIP_CL._ One potential factor could be the presence of airflow obstruction at the lower airway level [27], although this appears unlikely since we excluded individuals with abnormal spirometry in our control group. However, obstruction could occur at the upper airway (e.g., nasal) level [28], which we did not assess. A possibility also exists that the pattern and/or level of inspiratory muscle recruitment differs between SNIP_CL_ and SNIP_OP_. This might also help to explain a closer correlation between SNIP_CL_ and MIP, since the two are similar in being "static" in nature compared to the more "dynamic" SNIP_OP_. The specific maneuver itself, i.e., sniff vs. Mueller, is also an important element in determining inspiratory muscle recruitment, with brief sniffs generally producing higher values of diaphragm activation and transdiaphragmatic pressure than the inspiratory maneuver employed for MIP [29, 30]. However, SNIP_OP_ may conversely generate lower pressures than static maneuvers due to shortening of inspiratory muscles and the attendant pressure-velocity relationship [31]. In a clinical context, these sources of variability are difficult to ascertain, but the tests are not interchangeable and should be viewed as complementary [12].

### Critique of methods

It should be noted that our study design contains several elements which accurately reflect the routine clinical evaluation of NMD patients but may also introduce increased variability in the measurements. For example, NMD patients were comprised of a heterogeneous group of diagnoses with different levels of weakness. In this regard, Terzi et al. previously reported much wider limits of agreement between SNIP_OP_ and MIP in myotonic dystrophy than in Duchenne muscular dystrophy [7]. In addition, our control group was a clinical one rather than being composed of entirely healthy volunteers, although it should be emphasized that all control group subjects had normal spirometry. The MIP was initiated from RV as per standard clinical practice and American Thoracic Society recommendations [12], whereas SNIP was measured at FRC according to the original description of the technique [4]. These lung volume differences would be expected to result in a small (less than 10 cmH_2_O) change in inspiratory force generation [24, 32], which is quite consistent with the average magnitude of MIP minus SNIP_CL_ differences found in our study. Given that the study subjects did not undergo any prior training period, one possible limitation of the current study might be insufficient learning of the procedure. However, this appears unlikely since results were similar regardless of which test was performed first. Finally, as noted above we did not objectively measure nasal resistance, which can also affect SNIP reliability [4, 28].

It is important to emphasize that although SNIP test result variability may have been increased by one or more of the above factors, our findings are likely more generalizable to real world clinical practice for the very same reasons. In addition, since technical measurement errors often underestimate but are very unlikely to overestimate muscle strength, respiratory muscle pressure generation is primarily used as a “rule out” test for muscle weakness. Accordingly, our study suggests that the use of SNIP_CL_ in this manner may help to prevent clinical misclassification of certain patients who might otherwise be considered as having significant inspiratory muscle weakness based on low values for either MIP or SNIP_OP_.

## Conclusions

The SNIP_CL_ maneuver produces values which are systematically higher than SNIP_OP_ and therefore likely represents a more useful test for ruling out inspiratory muscle weakness. Accordingly, we propose that whenever MIP is low or cannot be performed, SNIP_CL_ should be used to obtain further information on inspiratory muscle strength. Clearly, the use of different tests of respiratory muscle strength should be considered complementary in nature as previously suggested by others [2, 3].

## Supporting information

S1 File Data file. Study data.
(XLS)
